# 3′-Benz­yloxy-3-hydr­oxy-3,3′-bi-1*H*-indole-2,2′(3*H*,3′*H*)-dione monohydrate

**DOI:** 10.1107/S1600536809012380

**Published:** 2009-04-08

**Authors:** P. Ramesh, S. S. Sundaresan, N. Vidhya Lakshmi, Paramasivan T. Perumal, M. N. Ponnuswamy

**Affiliations:** a Department of Physics, Presidency College (Autonomous), Chennai 600 005, India; b Centre of Advanced Study in Crystallography and Biophysics, University of Madras, Guindy Campus, Chennai 600 025, India; c Organic Chemistry Division, Central Leather Research Institute, Adyar, Chennai 600 020, India

## Abstract

In the title compound, C_23_H_18_N_2_O_4_·H_2_O, the two oxindole rings subtend a dihedral angle of 54.29 (5)°. The crystal structure is stabilized by intermolecular N—H⋯O, O—H⋯O and C—H⋯π inter­actions.

## Related literature

For the biological activity and pharmaceutical applications of indole derivatives, see: Harris & Uhle (1960 ▶); Ho *et al.* (1986 ▶); Rajeswaran *et al.* (1999 ▶); Stevenson *et al.* (2000 ▶). For description of hydrogen-bond motifs, see Bernstein *et al.* (1995 ▶).
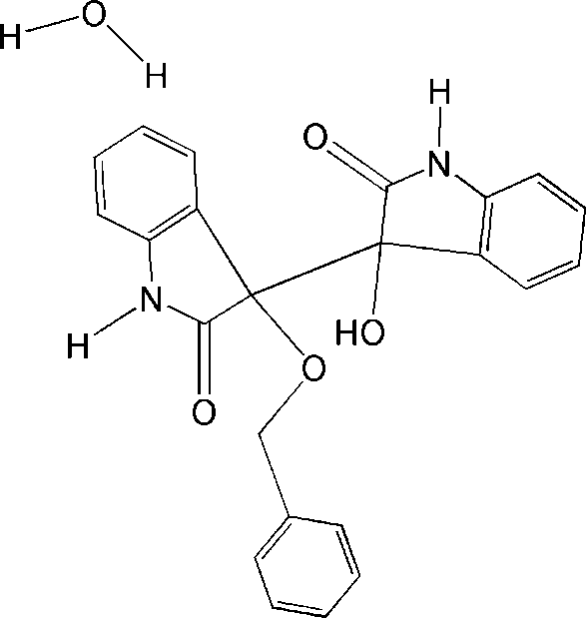


## Experimental

### 

#### Crystal data

C_23_H_18_N_2_O_4_·H_2_O
*M* *_r_* = 404.41
Triclinic, 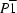

*a* = 9.8243 (3) Å
*b* = 9.9304 (6) Å
*c* = 11.4460 (5) Å
α = 107.517 (2)°
β = 114.227 (3)°
γ = 93.918 (2)°
*V* = 947.17 (8) Å^3^
*Z* = 2
Mo *K*α radiation
μ = 0.10 mm^−1^
*T* = 293 K
0.28 × 0.25 × 0.20 mm

#### Data collection

Bruker Kappa APEXII area-detector diffractometer
Absorption correction: multi-scan (*SADABS*; Sheldrick, 2001 ▶) *T*_min_ = 0.972, *T*_max_ = 0.980
16894 measured reflections
3335 independent reflections
3045 reflections with *I* > 2σ(*I*)
*R*_int_ = 0.021

#### Refinement

*R*[*F* ^2^ > 2σ(*F* ^2^)] = 0.032
*wR*(*F* ^2^) = 0.084
*S* = 1.05
3335 reflections
292 parameters
H atoms treated by a mixture of independent and constrained refinement
Δρ_max_ = 0.23 e Å^−3^
Δρ_min_ = −0.18 e Å^−3^

### 

Data collection: *APEX2* (Bruker, 2004 ▶); cell refinement: *SAINT* (Bruker, 2004 ▶); data reduction: *SAINT*; program(s) used to solve structure: *SHELXS97* (Sheldrick, 2008 ▶); program(s) used to refine structure: *SHELXL97* (Sheldrick, 2008 ▶); molecular graphics: *ORTEP-3* (Farrugia, 1997 ▶); software used to prepare material for publication: *SHELXL97* and *PLATON* (Spek, 2009 ▶).

## Supplementary Material

Crystal structure: contains datablocks global, I. DOI: 10.1107/S1600536809012380/bt2889sup1.cif

Structure factors: contains datablocks I. DOI: 10.1107/S1600536809012380/bt2889Isup2.hkl

Additional supplementary materials:  crystallographic information; 3D view; checkCIF report

## Figures and Tables

**Table 1:** Hydrogen-bond geometry (Å, °)

*D*—H⋯*A*	*D*—H	H⋯*A*	*D*⋯*A*	*D*—H⋯*A*
N1—H1⋯O5^i^	0.860 (18)	2.045 (18)	2.8858 (17)	165.6 (15)
O2—H2⋯O3^ii^	0.91 (2)	1.99 (2)	2.8463 (13)	155.9 (18)
O2—H2⋯O2^ii^	0.91 (2)	2.57 (2)	3.0274 (18)	111.9 (14)
O5—H5*B*⋯O3^iii^	0.96 (3)	1.97 (3)	2.8969 (17)	161 (3)
N12—H12⋯O1^iv^	0.877 (16)	2.044 (16)	2.8329 (14)	149.2 (14)
C17—H17⋯*Cg*3	0.93	3.15	3.8226 (17)	130
C16—H16⋯*Cg*5^v^	0.93	2.75	3.5140 (19)	140

Symmetry codes: (i) 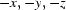; (ii) 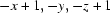; (iii) 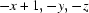; (iv) 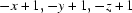; (v) 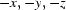. *Cg*3 is the centroid of the C2–C7 ring and *Cg*5 is the centroid of the C20–C25 ring.

**Fig. 1.**
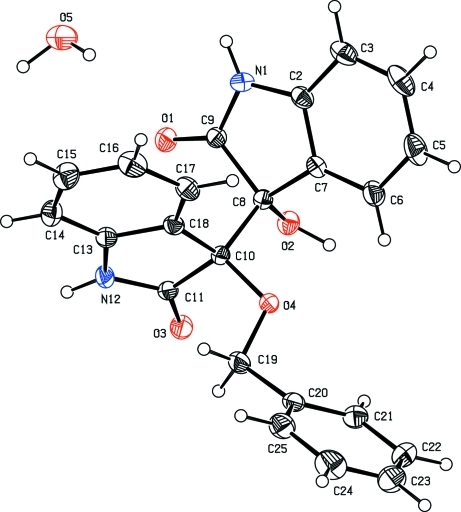
Perspective view of the molecule with displacement ellipsoids drawn at the 50% probability level. The H atoms are shown as small circles of arbitrary radii.

**Fig. 2.**
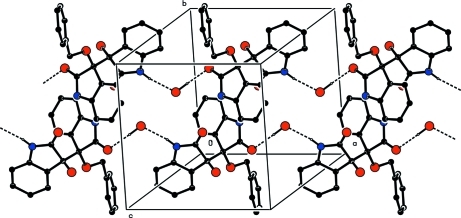
The crystal packing of the molecules viewed down c axis. H atoms not involved in hydrogen bonding have been omitted for clarity.

## supplementary crystallographic information

### Comment

Indole derivatives are used as bioactive drugs (Stevenson *et al.*, 2000) and they exibit anti-allergic, central nervous system depressant and muscle relaxant properties (Harris & Uhle 1960; Ho *et al.*, 1986). Indoles have been proved to display high aldose reductase inhibitory activity (Rajeswaran *et al.*,  1999).  Against this background and to ascertain the molecular conformation, the structure determination of the title compound has been carried out.

The ORTEP plot of the molecule is shown in Fig. 1. The oxindole rings enclose a dihedral angle of 54.29 (5)°. In the benzene ring of the indole ring system, the endocyclic angels at C3 and C14 are contracted to 117.24 (25)° and 116.90 (14)°, while those at C2 and C13 are expanded to 122.20 (14)° and 122.69 (13)°, respectively. This would appear to be a real effect caused by the fusion of the pyrrole with benzene ring resulting in an angular distortion. The sum of the bond angles around the hetero nitrogen atom in the oxindole ring systems are equal to N1 [359.76 (11)°] and N12 [359.93 (10)°], is in accordance with the *sp^2^* hybridization.

The packing of the molecules in the crystal structure is stabilized through  N-H···O, O-H···O and C-H···π interactions.  Atom N12 (x, y, z) donates a proton to O1 (-x+1, -y+1, -z+1) and forms a dimer with graph-set motif of R^2^_2_(14) (Bernstein *et al.,* 1995). The intermolecular N1-H1···O5 and O5-H5B···O3 hydrogen bonds form a one dimensional chain running along a-axis. The indole ring interacts with the other indole moiety through a weak intra C-H···π interaction involving atom C17, the separation between H17 and the centroid of the C2/C3/C4/C5/C6/C7 (Cg3) ring being 3.15Å.

### Experimental

To a refluxing solution of isatin (1 equivalent), Rh_2_(OAC)_4_ (0.01 equivalent) in dichloromethane, benzyl alcohol (1.2 equivalent) was added. After five minutes, 3-diazo-1,3-dihydro-indol-2-one in dichloromethane was added dropwise through a syringe pump. The reaction mixture was allowed to stir at 60°C for half-an-hour. The solid formed in the reaction was filtered and washed with methanol to obtain the pure product. The compound was recrystallized in ethanol.

### Refinement

H atoms bonded to nitrogen and oxygen H atoms were freely refined.  H atoms bonded to carbon were positioned geometrically (C—H=0.93–0.97 Å) and allowed to ride on their parent atoms  with 1.2*U*_eq_(C).

### Crystal data

**Table:**

C_23_H_18_N_2_O_4_·H_2_O	*Z* = 2
*M**_r_* = 404.41	*F*(000) = 424
Triclinic, *P*1	*D*_x_ = 1.418 Mg m^−^^3^
Hall symbol: -P 1	Mo *K*α radiation, λ = 0.71073 Å
*a* = 9.8243 (3) Å	Cell parameters from 4635 reflections
*b* = 9.9304 (6) Å	θ = 2.1–25.0°
*c* = 11.4460 (5) Å	µ = 0.10 mm^−^^1^
α = 107.517 (2)°	*T* = 293 K
β = 114.227 (3)°	Block, colourless
γ = 93.918 (2)°	0.28 × 0.25 × 0.20 mm
*V* = 947.17 (8) Å^3^	

### Data collection

**Table:**

Bruker Kappa APEXII area-detector diffractometer	3335 independent reflections
Radiation source: fine-focus sealed tube	3045 reflections with *I* > 2σ(*I*)
graphite	*R*_int_ = 0.021
ω and φ scans	θ_max_ = 25.0°, θ_min_ = 2.1°
Absorption correction: multi-scan (*SADABS*; Sheldrick, 2001)	*h* = −11→11
*T*_min_ = 0.972, *T*_max_ = 0.980	*k* = −11→11
16894 measured reflections	*l* = −13→13

### Refinement

**Table:**

Refinement on *F*^2^	Secondary atom site location: difference Fourier map
Least-squares matrix: full	Hydrogen site location: inferred from neighbouring sites
*R*[*F*^2^ > 2σ(*F*^2^)] = 0.032	H atoms treated by a mixture of independent and constrained refinement
*wR*(*F*^2^) = 0.084	*w* = 1/[σ^2^(*F*_o_^2^) + (0.038*P*)^2^ + 0.3004*P*] where *P* = (*F*_o_^2^ + 2*F*_c_^2^)/3
*S* = 1.05	(Δ/σ)_max_ = 0.003
3335 reflections	Δρ_max_ = 0.23 e Å^−^^3^
292 parameters	Δρ_min_ = −0.18 e Å^−^^3^
0 restraints	Extinction correction: *SHELXL*, Fc^*^=kFc[1+0.001xFc^2^λ^3^/sin(2θ)]^-1/4^
Primary atom site location: structure-invariant direct methods	Extinction coefficient: 0.038 (3)

### Special details

**Table:**

Geometry. All e.s.d.'s (except the e.s.d. in the dihedral angle between two l.s. planes) are estimated using the full covariance matrix. The cell e.s.d.'s are taken into account individually in the estimation of e.s.d.'s in distances, angles and torsion angles; correlations between e.s.d.'s in cell parameters are only used when they are defined by crystal symmetry. An approximate (isotropic) treatment of cell e.s.d.'s is used for estimating e.s.d.'s involving l.s. planes.
Refinement. Refinement of *F*^2^ against ALL reflections. The weighted *R*-factor *wR* and goodness of fit *S* are based on *F*^2^, conventional *R*-factors *R* are based on *F*, with *F* set to zero for negative *F*^2^. The threshold expression of *F*^2^ > σ(*F*^2^) is used only for calculating *R*-factors(gt) *etc*. and is not relevant to the choice of reflections for refinement. *R*-factors based on *F*^2^ are statistically about twice as large as those based on *F*, and *R*- factors based on ALL data will be even larger.

### Fractional atomic coordinates and isotropic or equivalent isotropic displacement parameters (Å^2^)

**Table:**

	*x*	*y*	*z*	*U*_iso_*/*U*_eq_	
O1	0.33868 (11)	0.37105 (9)	0.58225 (10)	0.0399 (2)	
O2	0.38716 (10)	0.07924 (10)	0.53420 (9)	0.0335 (2)	
H2	0.381 (2)	−0.018 (2)	0.504 (2)	0.070 (6)*	
O3	0.61939 (10)	0.21310 (10)	0.48237 (10)	0.0374 (2)	
O4	0.33920 (10)	0.00510 (8)	0.25071 (9)	0.0302 (2)	
O5	0.09912 (16)	−0.40448 (16)	−0.55511 (16)	0.0632 (3)	
H5B	0.183 (4)	−0.346 (3)	−0.552 (3)	0.129 (10)*	
H5A	0.064 (4)	−0.482 (4)	−0.622 (3)	0.135 (12)*	
N1	0.09503 (13)	0.23295 (12)	0.47470 (12)	0.0349 (3)	
H1	0.0522 (19)	0.2945 (18)	0.5098 (17)	0.048 (4)*	
C2	0.02036 (14)	0.08934 (14)	0.38791 (13)	0.0320 (3)	
C3	−0.12940 (15)	0.02332 (17)	0.34058 (15)	0.0435 (3)	
H3	−0.1961	0.0735	0.3647	0.052*	
C4	−0.17671 (17)	−0.12064 (19)	0.25573 (16)	0.0520 (4)	
H4	−0.2777	−0.1681	0.2211	0.062*	
C5	−0.07767 (19)	−0.19539 (17)	0.22128 (17)	0.0521 (4)	
H5	−0.1129	−0.2921	0.1632	0.062*	
C6	0.07409 (17)	−0.12838 (15)	0.27202 (14)	0.0407 (3)	
H6	0.1416	−0.1794	0.2502	0.049*	
C7	0.12222 (14)	0.01554 (13)	0.35540 (12)	0.0288 (3)	
C8	0.27766 (13)	0.11694 (12)	0.43061 (12)	0.0262 (3)	
C9	0.24378 (14)	0.25831 (13)	0.50590 (12)	0.0286 (3)	
C10	0.33824 (13)	0.14368 (12)	0.33172 (12)	0.0255 (3)	
C11	0.50226 (13)	0.24303 (12)	0.41187 (12)	0.0273 (3)	
N12	0.49318 (12)	0.36457 (11)	0.38326 (11)	0.0305 (2)	
H12	0.5721 (18)	0.4358 (17)	0.4159 (16)	0.042 (4)*	
C13	0.34446 (14)	0.36220 (13)	0.29098 (12)	0.0289 (3)	
C14	0.29438 (16)	0.46934 (14)	0.24218 (14)	0.0382 (3)	
H14	0.3602	0.5570	0.2701	0.046*	
C15	0.14143 (18)	0.44063 (16)	0.14958 (15)	0.0448 (4)	
H15	0.1036	0.5108	0.1147	0.054*	
C16	0.04399 (16)	0.31071 (17)	0.10790 (15)	0.0443 (4)	
H16	−0.0580	0.2940	0.0447	0.053*	
C17	0.09647 (15)	0.20413 (15)	0.15937 (13)	0.0359 (3)	
H17	0.0307	0.1164	0.1314	0.043*	
C18	0.24816 (14)	0.23153 (13)	0.25278 (12)	0.0277 (3)	
C19	0.40617 (18)	0.00789 (15)	0.16166 (15)	0.0418 (3)	
H19A	0.5160	0.0441	0.2151	0.050*	
H19B	0.3649	0.0719	0.1131	0.050*	
C20	0.37193 (14)	−0.14144 (13)	0.06125 (13)	0.0315 (3)	
C21	0.39451 (15)	−0.25784 (15)	0.10387 (14)	0.0370 (3)	
H21	0.4291	−0.2440	0.1963	0.044*	
C22	0.36598 (19)	−0.39435 (16)	0.00986 (17)	0.0492 (4)	
H22	0.3802	−0.4725	0.0390	0.059*	
C23	0.3165 (2)	−0.41584 (17)	−0.12678 (18)	0.0573 (4)	
H23	0.2985	−0.5080	−0.1897	0.069*	
C24	0.2940 (2)	−0.30139 (19)	−0.16987 (16)	0.0566 (4)	
H24	0.2607	−0.3157	−0.2623	0.068*	
C25	0.32036 (17)	−0.16459 (17)	−0.07653 (15)	0.0440 (3)	
H25	0.3033	−0.0874	−0.1067	0.053*	

### Atomic displacement parameters (Å^2^)

**Table:**

	*U*^11^	*U*^22^	*U*^33^	*U*^12^	*U*^13^	*U*^23^
O1	0.0466 (6)	0.0270 (5)	0.0427 (5)	0.0014 (4)	0.0243 (5)	0.0037 (4)
O2	0.0331 (5)	0.0333 (5)	0.0335 (5)	0.0095 (4)	0.0121 (4)	0.0154 (4)
O3	0.0245 (5)	0.0340 (5)	0.0483 (6)	0.0061 (4)	0.0110 (4)	0.0158 (4)
O4	0.0346 (5)	0.0230 (4)	0.0360 (5)	0.0038 (3)	0.0224 (4)	0.0061 (4)
O5	0.0581 (8)	0.0594 (8)	0.0800 (9)	0.0163 (6)	0.0439 (7)	0.0168 (7)
N1	0.0346 (6)	0.0360 (6)	0.0414 (6)	0.0138 (5)	0.0235 (5)	0.0135 (5)
C2	0.0298 (6)	0.0400 (7)	0.0316 (6)	0.0074 (5)	0.0149 (5)	0.0187 (6)
C3	0.0279 (7)	0.0621 (9)	0.0482 (8)	0.0095 (6)	0.0179 (6)	0.0292 (7)
C4	0.0302 (7)	0.0672 (11)	0.0510 (9)	−0.0090 (7)	0.0111 (7)	0.0258 (8)
C5	0.0502 (9)	0.0427 (8)	0.0482 (9)	−0.0148 (7)	0.0180 (7)	0.0082 (7)
C6	0.0428 (8)	0.0333 (7)	0.0433 (8)	−0.0011 (6)	0.0219 (7)	0.0089 (6)
C7	0.0282 (6)	0.0306 (6)	0.0293 (6)	0.0026 (5)	0.0139 (5)	0.0125 (5)
C8	0.0256 (6)	0.0250 (6)	0.0283 (6)	0.0057 (5)	0.0123 (5)	0.0096 (5)
C9	0.0332 (7)	0.0277 (6)	0.0296 (6)	0.0076 (5)	0.0174 (5)	0.0117 (5)
C10	0.0247 (6)	0.0217 (6)	0.0288 (6)	0.0041 (4)	0.0132 (5)	0.0064 (5)
C11	0.0257 (6)	0.0253 (6)	0.0304 (6)	0.0055 (5)	0.0145 (5)	0.0070 (5)
N12	0.0265 (5)	0.0251 (5)	0.0359 (6)	0.0003 (4)	0.0123 (5)	0.0096 (4)
C13	0.0311 (6)	0.0290 (6)	0.0279 (6)	0.0066 (5)	0.0152 (5)	0.0095 (5)
C14	0.0458 (8)	0.0319 (7)	0.0389 (7)	0.0085 (6)	0.0185 (6)	0.0164 (6)
C15	0.0505 (9)	0.0480 (8)	0.0432 (8)	0.0204 (7)	0.0189 (7)	0.0272 (7)
C16	0.0328 (7)	0.0612 (9)	0.0389 (8)	0.0134 (7)	0.0110 (6)	0.0247 (7)
C17	0.0290 (7)	0.0431 (7)	0.0334 (7)	0.0037 (6)	0.0119 (5)	0.0151 (6)
C18	0.0289 (6)	0.0293 (6)	0.0269 (6)	0.0061 (5)	0.0152 (5)	0.0092 (5)
C19	0.0520 (8)	0.0333 (7)	0.0501 (8)	0.0037 (6)	0.0375 (7)	0.0089 (6)
C20	0.0287 (6)	0.0326 (7)	0.0353 (7)	0.0053 (5)	0.0194 (5)	0.0084 (5)
C21	0.0376 (7)	0.0400 (7)	0.0350 (7)	0.0105 (6)	0.0179 (6)	0.0129 (6)
C22	0.0611 (10)	0.0361 (8)	0.0614 (10)	0.0175 (7)	0.0380 (8)	0.0161 (7)
C23	0.0735 (11)	0.0402 (8)	0.0540 (10)	0.0033 (8)	0.0412 (9)	−0.0039 (7)
C24	0.0673 (11)	0.0638 (11)	0.0328 (8)	0.0027 (8)	0.0273 (8)	0.0056 (7)
C25	0.0499 (9)	0.0494 (8)	0.0409 (8)	0.0117 (7)	0.0253 (7)	0.0201 (7)

### Geometric parameters (Å, °)

**Table:**

O1—C9	1.2191 (15)	N12—C13	1.4016 (16)
O2—C8	1.4066 (14)	N12—H12	0.877 (16)
O2—H2	0.91 (2)	C13—C14	1.3747 (18)
O3—C11	1.2218 (15)	C13—C18	1.3861 (17)
O4—C10	1.4123 (14)	C14—C15	1.384 (2)
O4—C19	1.4273 (15)	C14—H14	0.9300
O5—H5B	0.96 (3)	C15—C16	1.376 (2)
O5—H5A	0.83 (3)	C15—H15	0.9300
N1—C9	1.3392 (17)	C16—C17	1.391 (2)
N1—C2	1.4017 (17)	C16—H16	0.9300
N1—H1	0.860 (18)	C17—C18	1.3783 (18)
C2—C3	1.3742 (19)	C17—H17	0.9300
C2—C7	1.3851 (18)	C19—C20	1.4906 (18)
C3—C4	1.381 (2)	C19—H19A	0.9700
C3—H3	0.9300	C19—H19B	0.9700
C4—C5	1.375 (2)	C20—C25	1.3808 (19)
C4—H4	0.9300	C20—C21	1.3816 (19)
C5—C6	1.387 (2)	C21—C22	1.377 (2)
C5—H5	0.9300	C21—H21	0.9300
C6—C7	1.3759 (18)	C22—C23	1.374 (2)
C6—H6	0.9300	C22—H22	0.9300
C7—C8	1.5086 (16)	C23—C24	1.366 (3)
C8—C9	1.5477 (16)	C23—H23	0.9300
C8—C10	1.5551 (16)	C24—C25	1.380 (2)
C10—C18	1.5114 (17)	C24—H24	0.9300
C10—C11	1.5608 (16)	C25—H25	0.9300
C11—N12	1.3418 (16)		
			
C8—O2—H2	110.3 (12)	C13—N12—H12	123.8 (10)
C10—O4—C19	113.84 (9)	C14—C13—C18	122.68 (12)
H5B—O5—H5A	112 (3)	C14—C13—N12	127.59 (12)
C9—N1—C2	111.86 (11)	C18—C13—N12	109.73 (11)
C9—N1—H1	124.2 (11)	C13—C14—C15	116.90 (13)
C2—N1—H1	123.7 (11)	C13—C14—H14	121.5
C3—C2—C7	122.20 (13)	C15—C14—H14	121.5
C3—C2—N1	127.96 (13)	C16—C15—C14	121.59 (13)
C7—C2—N1	109.82 (11)	C16—C15—H15	119.2
C2—C3—C4	117.24 (14)	C14—C15—H15	119.2
C2—C3—H3	121.4	C15—C16—C17	120.67 (13)
C4—C3—H3	121.4	C15—C16—H16	119.7
C5—C4—C3	121.41 (13)	C17—C16—H16	119.7
C5—C4—H4	119.3	C18—C17—C16	118.49 (12)
C3—C4—H4	119.3	C18—C17—H17	120.8
C4—C5—C6	120.82 (14)	C16—C17—H17	120.8
C4—C5—H5	119.6	C17—C18—C13	119.65 (12)
C6—C5—H5	119.6	C17—C18—C10	131.70 (11)
C7—C6—C5	118.33 (14)	C13—C18—C10	108.63 (10)
C7—C6—H6	120.8	O4—C19—C20	109.22 (10)
C5—C6—H6	120.8	O4—C19—H19A	109.8
C6—C7—C2	119.98 (12)	C20—C19—H19A	109.8
C6—C7—C8	131.53 (12)	O4—C19—H19B	109.8
C2—C7—C8	108.41 (10)	C20—C19—H19B	109.8
O2—C8—C7	114.09 (10)	H19A—C19—H19B	108.3
O2—C8—C9	105.88 (9)	C25—C20—C21	118.92 (12)
C7—C8—C9	101.76 (9)	C25—C20—C19	119.93 (13)
O2—C8—C10	111.95 (9)	C21—C20—C19	121.13 (12)
C7—C8—C10	112.73 (9)	C22—C21—C20	120.20 (13)
C9—C8—C10	109.61 (9)	C22—C21—H21	119.9
O1—C9—N1	126.95 (12)	C20—C21—H21	119.9
O1—C9—C8	124.94 (11)	C23—C22—C21	120.39 (15)
N1—C9—C8	108.10 (10)	C23—C22—H22	119.8
O4—C10—C18	115.50 (9)	C21—C22—H22	119.8
O4—C10—C8	105.35 (9)	C24—C23—C22	119.82 (14)
C18—C10—C8	112.18 (9)	C24—C23—H23	120.1
O4—C10—C11	110.65 (9)	C22—C23—H23	120.1
C18—C10—C11	101.56 (9)	C23—C24—C25	120.11 (14)
C8—C10—C11	111.77 (9)	C23—C24—H24	119.9
O3—C11—N12	125.87 (11)	C25—C24—H24	119.9
O3—C11—C10	126.42 (11)	C24—C25—C20	120.55 (14)
N12—C11—C10	107.61 (10)	C24—C25—H25	119.7
C11—N12—C13	112.33 (10)	C20—C25—H25	119.7
C11—N12—H12	123.8 (10)		
			
C9—N1—C2—C3	176.59 (13)	O4—C10—C11—O3	−50.44 (16)
C9—N1—C2—C7	−2.02 (15)	C18—C10—C11—O3	−173.59 (12)
C7—C2—C3—C4	−1.4 (2)	C8—C10—C11—O3	66.64 (15)
N1—C2—C3—C4	−179.82 (13)	O4—C10—C11—N12	126.13 (10)
C2—C3—C4—C5	0.7 (2)	C18—C10—C11—N12	2.99 (12)
C3—C4—C5—C6	0.6 (2)	C8—C10—C11—N12	−116.79 (11)
C4—C5—C6—C7	−1.3 (2)	O3—C11—N12—C13	175.33 (12)
C5—C6—C7—C2	0.7 (2)	C10—C11—N12—C13	−1.27 (13)
C5—C6—C7—C8	177.20 (13)	C11—N12—C13—C14	178.62 (12)
C3—C2—C7—C6	0.65 (19)	C11—N12—C13—C18	−1.22 (14)
N1—C2—C7—C6	179.35 (11)	C18—C13—C14—C15	−1.10 (19)
C3—C2—C7—C8	−176.58 (11)	N12—C13—C14—C15	179.07 (12)
N1—C2—C7—C8	2.12 (14)	C13—C14—C15—C16	−0.2 (2)
C6—C7—C8—O2	−64.69 (17)	C14—C15—C16—C17	0.8 (2)
C2—C7—C8—O2	112.10 (11)	C15—C16—C17—C18	−0.1 (2)
C6—C7—C8—C9	−178.23 (13)	C16—C17—C18—C13	−1.14 (19)
C2—C7—C8—C9	−1.44 (12)	C16—C17—C18—C10	176.85 (12)
C6—C7—C8—C10	64.46 (17)	C14—C13—C18—C17	1.79 (19)
C2—C7—C8—C10	−118.75 (11)	N12—C13—C18—C17	−178.35 (11)
C2—N1—C9—O1	−177.54 (12)	C14—C13—C18—C10	−176.62 (11)
C2—N1—C9—C8	1.01 (14)	N12—C13—C18—C10	3.23 (13)
O2—C8—C9—O1	59.33 (15)	O4—C10—C18—C17	58.38 (17)
C7—C8—C9—O1	178.85 (11)	C8—C10—C18—C17	−62.37 (16)
C10—C8—C9—O1	−61.60 (15)	C11—C10—C18—C17	178.15 (13)
O2—C8—C9—N1	−119.26 (10)	O4—C10—C18—C13	−123.47 (11)
C7—C8—C9—N1	0.27 (12)	C8—C10—C18—C13	115.79 (11)
C10—C8—C9—N1	119.82 (11)	C11—C10—C18—C13	−3.70 (12)
C19—O4—C10—C18	61.51 (14)	C10—O4—C19—C20	−168.10 (10)
C19—O4—C10—C8	−174.11 (10)	O4—C19—C20—C25	133.58 (13)
C19—O4—C10—C11	−53.14 (13)	O4—C19—C20—C21	−48.07 (17)
O2—C8—C10—O4	73.14 (11)	C25—C20—C21—C22	0.1 (2)
C7—C8—C10—O4	−57.10 (12)	C19—C20—C21—C22	−178.29 (13)
C9—C8—C10—O4	−169.68 (9)	C20—C21—C22—C23	0.7 (2)
O2—C8—C10—C18	−160.41 (9)	C21—C22—C23—C24	−0.7 (3)
C7—C8—C10—C18	69.34 (12)	C22—C23—C24—C25	−0.1 (3)
C9—C8—C10—C18	−43.23 (13)	C23—C24—C25—C20	1.0 (3)
O2—C8—C10—C11	−47.09 (13)	C21—C20—C25—C24	−0.9 (2)
C7—C8—C10—C11	−177.34 (9)	C19—C20—C25—C24	177.44 (14)
C9—C8—C10—C11	70.09 (12)		

### Hydrogen-bond geometry (Å, °)

**Table:**

*D*—H···*A*	*D*—H	H···*A*	*D*···*A*	*D*—H···*A*
N1—H1···O5^i^	0.860 (18)	2.045 (18)	2.8858 (17)	165.6 (15)
O2—H2···O3^ii^	0.91 (2)	1.99 (2)	2.8463 (13)	155.9 (18)
O2—H2···O2^ii^	0.91 (2)	2.57 (2)	3.0274 (18)	111.9 (14)
O5—H5B···O3^iii^	0.96 (3)	1.97 (3)	2.8969 (17)	161 (3)
N12—H12···O1^iv^	0.877 (16)	2.044 (16)	2.8329 (14)	149.2 (14)
C17—H17···Cg3	0.93	3.15	3.8226 (17)	131
C16—H16···Cg5^v^	0.93	2.75	3.5140 (19)	140

Symmetry codes: (i) −*x*, −*y*, −*z*; (ii) −*x*+1, −*y*, −*z*+1; (iii) −*x*+1, −*y*, −*z*; (iv) −*x*+1, −*y*+1, −*z*+1;  (v) −*x*, −*y*, −*z*.

## References

[bb1] Bernstein, J., Davis, R. E., Shimoni, L. & Chang, N. L. (1995). *Angew. Chem. Int. Ed. Engl.* **34**, 1555–1573.

[bb2] Bruker (2004). *APEX2* and *SAINT* Bruker AXS Inc., Madison, Wisconsin, USA.

[bb3] Farrugia, L. J. (1997). *J. Appl. Cryst.* **30**, 565.

[bb4] Harris, L. S. & Uhle, F. C. (1960). *J. Pharmacol. Exp. Ther.* **128**, 353-363. 14399979

[bb5] Ho, C. Y., Haegman, W. E. & Perisco, F. (1986). *J. Med. Chem.* **29**, 118-121.

[bb6] Rajeswaran, W. G., Labroo, R. B., Cohen, L. A. & King, M. M. (1999). *J. Org. Chem.* **64**, 1369-1371.

[bb7] Sheldrick, G. M. (2001). *SADABS* University of Göttingen, Germany.

[bb8] Sheldrick, G. M. (2008). *Acta Cryst.* A**64**, 112–122. 10.1107/S0108767307043930 18156677

[bb9] Spek, A. L. (2009). *Acta Cryst.* D**65**, 148–155. 10.1107/S090744490804362X PMC2631630 19171970

[bb10] Stevenson, G. I., Smith, A. L., Lewis, S. G., Neduvelil, J. G., Patel, S., Marwood, R. & Castro, J. L. (2000). *Bioorg. Med. Chem. Lett.* **10**, 2697-2704. 10.1016/s0960-894x(00)00557-6 11133071

